# Fabricating of high-performance functional graphene fibers for micro-capacitive energy storage

**DOI:** 10.1038/srep29534

**Published:** 2016-07-08

**Authors:** Tianju Fan, Chunyan Zhao, Zhuangqing Xiao, Fangjun Guo, Kaiyu Cai, Hai Lin, Yidong Liu, Hong Meng, Yong Min, Arthur J. Epstein

**Affiliations:** 1 School of Advanced Materials, Peking University Shenzhen Graduate School, Shenzhen 518055, China; 2 Institute of Advanced Materials, Nanjing University of Posts and Telecommunications, Nanjing 210046, China; 3 Department of Physics and Chemistry & Biochemistry, Ohio State University, Columbus, OH 43210, USA

## Abstract

Although graphene is a typical two dimensional materials, it has converted to multi-dimensional materials with many unique properties. As an example, the one dimensional graphene fiber is fabricated by utilizing ionic liquid as coagulation and functional diamines as cross-linkers to connect graphene oxide layers. The fibers show excellent mechanical properties and superior electrical performance. The tensile strength of the resultant fibers reaches ~729 MPa after a super high temperature thermal annealing treatment at 2800 °C. Additionally, quasi-solid-state flexible micro-capacitors are fabricated with promising result on energy storage. The device show a specific volumetric capacity as high as ~225 F/cm^3^ (measured at 103.5 mA cm^−3^ in a three-electrode cell), as well as a long cycle life of 2000 times. The initial results indicate that these fibers will be a good candidate to replace energy storage devices for miniaturized portable electronic applications.

As a two-dimensional monolayer of carbon atoms with honeycomb lattice, graphene has been touted as a candidate for a variety of applications in the areas of structural materials, energy storage, electronic devices, etc.^1^,^2^,^3^. The excellent mechanical, electrical, and thermal properties of graphene compared with those of other reported materials have been observed in single-layer or multilayer graphene synthesized through chemical vapour deposition or epitaxial growth methods^2^,^4^. However, graphene can be assembled into various interesting structures, including zero-dimensional quantum dots, one-dimensional nanoscrolls or fibers, two dimensional films as well as three-dimensional foams^5^,^6^,^7^,^8^. In general, graphene based materials have demonstrated high gravimetric capacitances but poor volumetric performances for the supercapacitor application. A possible explanation of this behaviour lies in the strong inter sheet π-π interaction. While increasing the packing density, high ion accessibility is not allowed^9^,^10^.

Recently, people are impressed dramatically by the mechanical performance enhancement of graphene fibers (GF) either are prepared by spinning method^11^,^12^ or hydrothermal method^13^. One typical approach is to obtain graphene fibers through solution spinning of a liquid crystalline type graphene oxide (GO) spinning solution into coagulation agents^14^. The advantage of the GO fiber (GOF) is that we can adjust their mechanical and electrical properties by modifying interlayer spaces through appropriate chemical or thermal treatments to optimize the GF^15^. This type GF is easily fabricated by the standard industrial solution spinning process at a cost effective way. Additionally, the abundant oxygen functional groups on GO sheets could be converted or reduced easily, which could help us to convert those fibers into multifunctional textiles for various potential applications in flexible sensors and electronic devices^16^,^17^.

A recent report^18^ indicates that coagulating agents can act as coordinative cross-linkers between the GOF and divalent ions (CaCl_2_ and CuSO_4_), which can partially reduce the GOF with hydroxide ions, or reduce the surface charges on the GOF by introducing positively charged molecules or polymers (hexadecyltrimethyl-ammonium bromide, CTAB; chitosan) onto the surfaces of the GO layers. However, these coagulating agents tested inevitably require the additional use of a postdrawing process, such as rotating the coagulation stage, to uniformly orient the GOFs and maximize the packing density of the graphene layers in the GOF^18^,^19^,^20^.

Micro-supercapacitors (micro-SCs) are promising alternatives as they can provide higher power densities, faster charge/discharge rates and much longer lifetimes^21^,^22^,^23^,^24^,^25^. However, the key challenge for their use in practical applications is how to increase their energy density to close and even exceed those of micro batteries without compromising other electrochemical characteristics^26^,^27^. Therefore, electrodes with large volumetric capacitance need to be developed.

This work describes the facile fabrication of GFs with excellent mechanical properties, fabricating GOF-a by utilizing ionic liquid as coagulant and GOF-b by exploiting diamine as cross-linkers in spinning process. The corresponding GF-a and GF-b are obtained by reducing at 600 °C under nitrogen condition. GOFs are also annealing at 1200 °C under N_2_ atmosphere, 2200 °C and 2800 °C under Ar atmosphere. The resultant fibers present high electrical and thermal conductivities, low density, excellent tensile strength and high specific volumetric capacity. Based on those GFs, quasi-solid-state flexible micro-capacitors devices are fabricated with excellent performance.

## Results and Discussion

Based on the wet-spinning fiber process from the GO solution, we have measured the dimensions and thickness of the large GO sheets to determine whether the synthesized GO exhibit liquid crystalline behaviour. The AFM image indicates that GO sheets have an average layer thickness of 1.2 nm and an average size of 5–20 μm (Figure S1). The aspect ratio is sufficiently high enough to allow the as-prepared aqueous GO solution to present liquid crystalline behavior^28^,^29^. But different from the prior report^28^,^29^, our GO solution’s concentration is not low enough to show the liquid crystalline behaviours with the largest area of uniform orientation.

**Figure 1:**
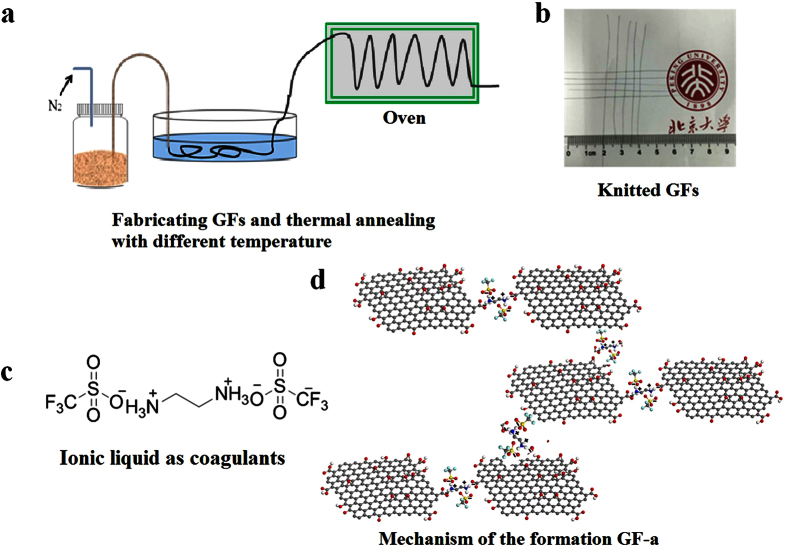
The fiber is fabricated by injecting a homogeneous GO solution and thermal annealing at 600 °C and 1200 °C in N_2_ atmosphere, 2200 °C and 2800 °C in Ar atmosphere. (**a**,**b**) The photos of a knitted textile fabricated from GF-a after annealing at 2800 °C. (**c**) Ionic liquid as coagulants in the process of spinning GOF. (**d**) Schematic illustrations of the formation non-covalent bond with ionic liquid between GO layers.

As illustrated from Fig. 1a, GOFs can be fabricated through an aqueous GO spinning solution under a N_2_ pressure controlled system. Those GOFs are strong enough to go through the coagulation bath, where the ionic liquid with organic solvent is used as a new coagulation. Several meters of the GOFs are rolled up with a taking-up spool, and then transformed them into an over for reducing and annealing treatment. The results indicate that the graphene layers could be easily aligned under shear forces as the GO solution passed through the capillary needle during the N_2_ pressured spinning process. Comparing to the direct wet-spun processing of reduced GO (rGO) fibers^30^, the GOF process is more rubbished and controllable. This is because the GOF has more alignment and stabilities in the presence of appropriate coagulating agents, which enhanced the interactions between the graphene layers. The final GFs are obtained by thermal reducing at 600 °C for 5 minutes or 1200 °C for 5 minutes under N_2_ atmosphere, 2200 °C for 5 minutes or 2800 °C for 5 minutes under Ar atmosphere. The resulted fibers are also flexible and can be bent and woven into textile structures (in Fig. 1b).

As shown the Figure S2a, the p-phenylenediamine is used as diamine coagulating agents to fabricate GF in a similar way. A schematic illustration of the formation covalent bond between diamines and GO layers is shown in Figure S2b. The GOFs are obtained by using the ionic liquid as coagulating agents (Fig. 1c,d) or diamines as cross-linkers during the spinning process. Here, the ionic liquid molecules act as coagulation agents by connecting those GO layers.

It is reported that bisamine along with carbon chain is used as coagulation agents through ionic bridges during the spinning process^31^,^32^. Several other coagulants included ammonium groups (-NH_3_^+^) or Ca^2+^ ions are also reported act as coagulation agents that shielded the negatively charged oxygen-containing acidic groups on the GO surfaces to weaken electrical repulsion and facilitate the assembly of GO layers into a fibrous shape^33^. Unfortunately, the counter ions along with these anions (Br^−^ or Cl^−^) tend to disrupt the packing efficient of the GO layers^8^,^19^. This is because the strong electrical attraction between ionic bridges and GO layers cannot be interrupted by counter ions. The GO layers prepared using the method described here display a high degree of alignment and packing order compared to that in the GOFs reported previously^28^,^34^. The alignment and packing order of the GOFs are further stabilized by the formation of covalent bonds between ionic liquid molecules and GO layers during the drying and thermal treatment processes.

However, the ionic bridges are also generated by rapid acid-base reactions between the functional groups on the GO layers and the basic amine functional groups when ionic liquid is utilizing by coagulation agents in the spinning process. Once the GO solution is immersed in the fiber spinning coagulation bath, the acidic groups, such as the ionic liquid on the GO layer, donated protons to the amine group on the linker, form a conjugated base and acid, respectively. The diamine formed a strong covalent cross-linker with GO sheet, while ionic liquid linker and the GO surface formed non-covalent ionic bridge as schematic illustrated in Fig. 1d and Figure S2. When diamine is used as coagulant, GOF-b is obtained rapidly. However, GOF-a is formed much slower when ionic liquid is used as coagulant in the spinning process.

**Figure 2:**
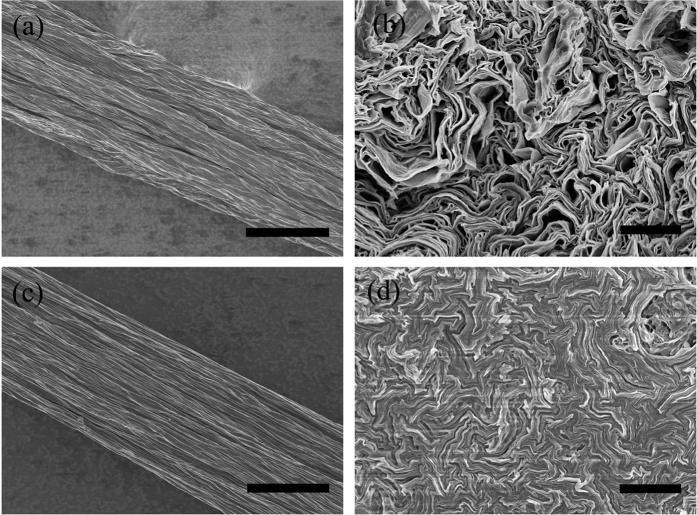
SEM images of the surfaces (**a**,**c**) and cross-sectional morphologies (**b**,**d**) of GOF-b and GOF-a, respectively. Scale bars: 50 μm (**a**,**c**), 500 nm (**b**,**d**).

Figure 2 showed the morphology of the surface and cross-sectional SEM images of the GOF-a and GOF-b where the ionic liquid and diamine are used as cross-linkers. The GO layers with ionic liquid cross-linker are well-aligned along the longitudinal spinning direction (Fig. 2a,b), and the layers of p-phenylenediamine are closely packed in a lamellar structure (Fig. 2c,d).

The SEM images of twisting GF-a to yarns GFs with two fibers are shown in the Figure S3. This type of lamellar structure has been reported previously in GOFs coagulated using other coagulants, such as CTAB or CaCl_2_^15^,^20^. The inner fiber structures of the GF-a and GF-b after annealing at 2800 °C are shown in Figures S4 and S5. The highly aligned structure in the pure graphene sheets is well maintained in the GF-a and GF-b before annealing at 2800 °C, and the annealed graphene sheets stack into a layer-by-layer structure throughout the cross section of GFs. However, microvoids are generated after thermal annealing at 2800 °C among graphene sheets resulting a higher porosity.

Figure S6 presented the FT-IR spectra of the GOF-a, GOF-b and GOF-c samples after the fibers drying process. GOF-a is supported by the observation of -CO-NH- (1720 cm^−1^) stretching and bending modes, which indicated the carboxylic groups ionized and formed ionic bridges with non-covalent bond. The GO layers are effectively cross-linked by both ionic bridge formation and covalent bonds during the spinning and drying processes, respectively. The resultant GOF-a and GOF-b have presented excellent mechanical properties.

**Figure 3:**
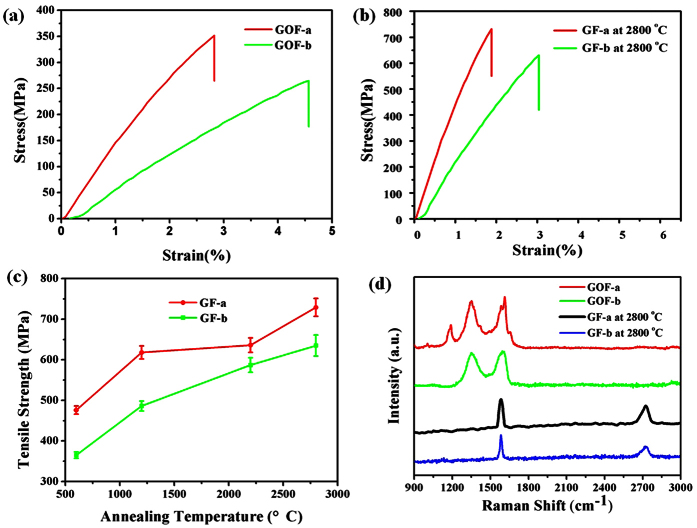
(**a**) Representative stress strain curves of the GOF-a, GOF-b sample at 25 °C; and (**b**) the stress strain curves of the GF-a, GF-b sample after annealing at 2800 °C; (**c**) tensile strength of GF-a and GF-b after annealing at different temperatures; (**d**) polarized Raman spectra from GOF-a, GOF-b, GF-a and GF-b after annealing 2800 °C.

Figure 3a show representative stress-strain curves of the GOFs prepared using cross-linked ionic liquid molecules and diamine molecules. The as-prepared dry hybrid GOF-a and GOF-b exhibit excellent mechanical performance, where the tensile strength of GOF-a and GOF-b are 356 ± 16 MPa and 296 ± 12 MPa, respectively. Those fibers have presented much better tensile strength comparing to those of wet-spun rGO fiber and CNTs^35^,^36^. Additionally, they are flexible and can be bent into different shapes or woven into textile structures.

The high-packing density and large ion-accessible surface area are two prerequisites for SC electrodes to achieve high volumetric performance^37^,^38^. Table 1 lists various physical performance in terms of the density, surface area, conductivity and tensile strength of GOFs and GFs at different annealing temperatures, which confirmed their potential uses at high-power electronics and high-performance composite materials. Additionally, the annealed GF-a and GF-b present exceptional well mechanical properties as measured by tensile testing in the Fig. 3b,c. The tensile strength of the annealed GF-a and GF-b after annealing at 2800 °C have reached 729 ± 21 MPa and 632 ± 26 MPa, respectively. It is found that the GF’s mechanical strength by using ionic liquid as crosslinking agent is much higher than the one by using diamine as crosslinking agent after the thermal annealing. The possible reason is due to the graphene interlayer interaction is primarily dominated by the van der waals force among those adjacent graphene sheets^39^.

Figure 3d presented the typical D and G bands of GOF-a and GOF-b. The integrated intensity ratios of the D band (1345 cm^−1^) and G band (1609 cm^−1^) are calculated. GOF-a shows higher I_D_/I_G_ value of 1.05, and GOF-b presents lower I_D_/I_G_ value of 0.98. The peak at 1150 cm^−1^ in the Raman Spectra of GOF-a represents nanocrystalline diamond structures^40^,^41^, which might be the reason of GOF-a shows better tensile strength than GOF-b. After annealing at 2800 °C, the D band of those fibers is completely disappear, the fibers are converting sp^2^ domains with less defects. Moreover, the intercalation of the graphene sheets leads to greater tensile strength.

**Figure 4:**
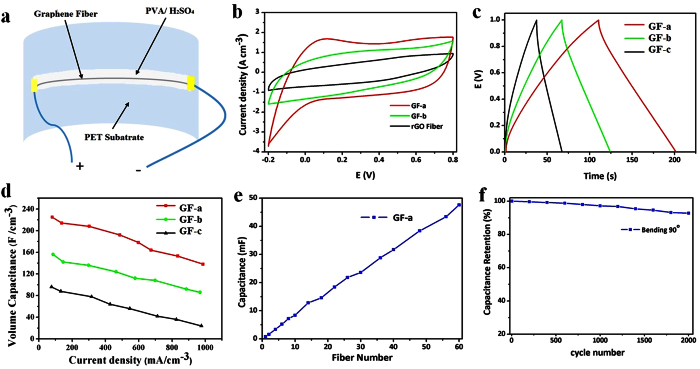
(**a**) Schematic of a micro-SC constructed using three fibers electrodes on polyester (PET) substrate. (**b**,**c)**. Cyclic voltammetry curves and Galvanostatic charge/discharge curves at various current densities of three fibers, GF-a, GF-b and GF-c, respectively. (**d**) Specific volumetric capacitance of the GF-a, GF-b and GF-c have measured in a three-electrode configuration in 1 M H_2_SO_4_. (**e**) Relation between total device capacitance and numbers of GF-a integrated. (**f)** Capacitance retention of GF-b after 2,000 cycles up to 90° bending angle.

We construct flexible micro-SCs as shown in the Fig. 4a by utilizing GF-a, GF-b and GF-c after annealing at 600 °C. Typically, two parallel GFs electrodes are mounted onto a flexible polyester (PET) substrate using PVA-H_2_SO_4_ as electrolyte without binder, current collector, separator or any packaging material^42^. The CV curves show reversible cathodic and anodic peaks at −0.2–0.8 V versus Ag/AgCl (Fig. 4b), indicating the presence of pseudocapacitance, suggesting that the pseudocapacitance is induced by oxygen-containing functional groups and/or nitrogen heteroatoms^42^. The enhanced capacitive performance induced by nitrogen doping is further confirmed by comparative CV curves of the fibers. The specific volumetric capacitance (Csp) of the fibers is calculated using their galvanostatic discharge curves (Fig. 4c,d). Its galvanostatic charge/discharge curves has a triangular shape, indicating excellent reversibility and good charge propagation between the two fibers electrodes. Figure 4c has illustrated that the volumetric capacitance of GF-a, GF-b and GF-c are 45.0 F cm^−3^ at 36 mA cm^−3^, 25.1 F cm^−3^ at 40 mA cm^−3^ and 18.1 F cm^−3^ at 28 mA cm^−3^, respectively. It is found that GF-a has the highest specific volumetric capacitance of 225 F cm^−3^ at 103.5 mA cm^−3^ in 1 M H_2_SO_4_ in the three-electrode cell. GF-b and GF-c demonstrated specific volumetric capacitance of 136 F cm^−3^ at 98 mA cm^−3^ and 96 F cm^−3^ at 101 mA cm^−3^, respectively. Moreover, as shown in Fig. 4e, assembled micro-SCs are constructed in parallel by incorporating a different number of fibers up to 60 fibers. The overall capacitance of the assembled micro-SCs increased linearly with the number of fibers increasing, showing good scalability.

**Figure 5:**
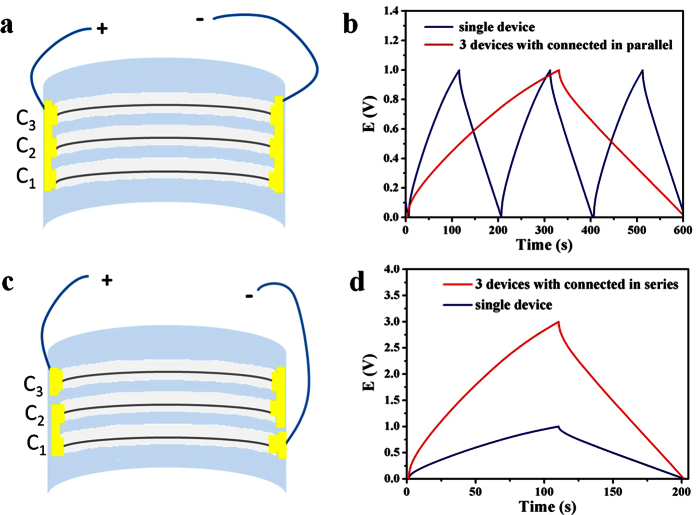
Assembly of microfibers in micro-SCs and their integration in a self-poared nanosystem. **(a**) Schematic and photograph of the three micro-SCs connected in parallel. (**b**) Galvanostatic charge/discharge curves of the device shown in (**a**). (**c**) Three micro-SCs connected in series. (**d**) Galvanostatic charge/discharge curves of the device shown in (**c**).

The micro-SC is further subjected to mechanical bending tests and the result is shown in Fig. 4f. It show negligible capacitance change that the micro-SC retains 93% of its initial capacitance with 90° bending after 2000 charge/discharge cycles, which demonstrate an impressive performance on stability as well as a long cycle life. This has demonstrated that a high flexibility and electrochemical stability are desirable perimeters for flexible electronics. Generally, comparing to the gravimetric power/energy density, the volumetric power/energy density of SCs is more important parameter for evaluating the energy storage performance of the micro-device by utilizing active electrode materials^43^. In order to meet specific energy and power needs for practical applications, three micro-SCs are assembled in parallel (Fig. 5a,b) and in series (Fig. 5c,d). The output current of the parallel assembled micro-SC increased by a factor of three and its discharge time is also three times that of a single assembled micro-SC when the operating current density remains the same (Fig. 5b). Comparing to a single micro-SC at an operating voltage of 1.0 V, the three assembled micro-SCs connected in series exhibit a 3.0 V charge/discharge voltage at the same discharge time (Fig. 5d).

## Conclusions

The GOFs can be easily prepared by increasing the electrical attraction between ionic liquid/diamine and GO layers without using the concern of liquid crystalline fabrication. Their corresponding GFs are formed through thermal reducing process with excellent mechanical properties. A super high temperature (up to 2800 °C) thermal annealing technique is applied to the GFs with significant performance enhancement. By utilizing those unique fibers, quasi-solid-state flexible micro-capacitors devices are constructed with excellent electrical performance. The resultant GFs as well as their micro-capacitors devices show high tensile strength, conductivity, and the specific volumetric capacity of GF-a is 225 F cm^−3^ at 104 mA cm^−3^ as well as a long cycle life up to 2000 times. These attractive results give us a new way to promote the GF properties by adjusting interlayer spaces through different coagulation agents and to construct micro electronic devices for the potential miniaturized portable electronic applications.

## Method

### Chemicals and materials

Graphite powder (200 mesh) is purchased from Baichuan Graphite Co. Ltd. (Qingdao, China); p-phenylenediamine is purchased from Aladdin; ionic liquid (1,2-ethylenediamino ditriflate), potassium permanganate (KMnO_4_), sodium nitrate (NaNO_3_), NaOH, sulfuric acid (H_2_SO_4_), hydrochloric acid (HCl), and hydrogen peroxide (H_2_O_2_, 30%) are all analytical grade and purchased from Shanghai Sinopharm Chemical Reagent Co., Ltd (Shanghai, China). All aqueous solutions are prepared with ultrapure water (18 MΩ). All regents are used without further purification.

### Preparation of GO

GO is prepared by the modified hummers method^44^. Briefly, 1.0 g of graphite and 60 mL of H_2_SO_4_ (98%) is stirred in an ice bath, 5.8 g of KMnO_4_ is added slowly with stirring for 0.5 h. The solution is heated to 30 °C for 2 h, 40 mL of deionizer water is added slowly, the mixture is then heated to 90 °C for 30 min, and 80 mL of deionizer water is added. When the temperature is cooled to 60 °C, 10 mL of H_2_O_2_ (30%) is added to give an orange yellow solution. 200 mL of 5% HCl solution is added, decanted the supernatant, and centrifuged with deionizer water to pH 4 to 6, the mixture solution is further dialyzed in a dialysis bag in 2 days, and then low density large GO is obtained.

### Preparation of GOF-a, GOF-b and GOF-c

The GOF-a, GOF-b and GOF-c are fabricated by using the coagulation bath of 0.1 M ionic liquid (1,2-ethylenediamino triflate) in water, 0.2 M p-phenylenediamine in ethanol, and 0.2 M NH_3_·H_2_O in water. During the spinning process for GO gel solution (10 mg/mL) are injected into different coagulation baths by using a spinning needle at a rate of 10 mL/min under 1.5 MPa held by N_2_. The as-spun GOF is wished with methanol and rolled up onto a collection spool or vertically aligned and dried at room temperature.

### Preparation of GF-a, GF-b and GF-c

GF-a, GF-b and GF-c are obtained by reducing GOF-a, GOF-b and GOF-c with annealing at 600 °C under N_2_ atmosphere. GOF-a and GOF-b are also annealing 5 min at 1200 °C under N_2_ atmosphere, 2200 °C and 2800 °C under Ar atmosphere, which are marked as GF-a or GF-b with certain temperature.

### Characterization and Electrochemical measurement

The as-produced GO is uniformly deposited onto an ozone-treated silicon substrate and characterized by scanning electron microscopy (SEM) (JEOL, JSM-4800) and atomic force microscopy (AFM) (Bruker, Fastscan) to analyze the dimensions of the single GO sheet. The surface and cross-sectional morphologies of each GOF are analyzed using SEM. The chemical states of the GOFs are investigated by examining the fibers using Fourier transform infrared spectroscopy (FT-IR) (Nicolet iS10, Thermo Scientific). The mechanical properties of the GOFs are characterized using a tensile test machine (Sust, WAW-300B) with gauge length of 1 cm and strain rate of 10%/min. The results reported are the average measured values obtained from at least 10 samples of each GOF. The specific surface area is analysed using Accelerated Surface Area and Porosimetry System (Micromeritics, ASAP 2020 HD88) and calculated through Brunauer-Emmett-Teller (BET) method. The densities of fibers are calculated from the mass and volume, and the volume is obtained by multiple the area of cross section and the length of fibers.

### Electrochemical characterization of individual fibers

A three-electrode cell, consisting of an Ag/AgCl (3 M KCl) electrode as the reference electrode, a platinum wire as the counter electrode and a single fiber as the working electrode in 1 M H_2_SO_4_ electrolyte, is used for capacitance measurements by a potentiostat (CHI 660E). C_electrode_ is calculated from the galvanostatic discharge curves, using the equation C_electrode_ = i/(dV/dt), where i is the discharge current, and dV/dt is the slope of the discharge curve.

### Fabrication and characterization of quasi-solid-state micro-SCs

The polymeric gel electrolyte (PVA/H_2_SO_4_) is prepared according to a previously reported method^45^. Two dry fibers with the same length are immersed in the PVA/H_2_SO_4_ electrolyte solution for 5 min. Then, the electrolyte wetted fibers are placed on a PET film, in parallel. Finally, the assembled device is dried under ambient conditions until the PVA/H_2_SO_4_ gel solidified. The performance of the assembled micro-SCs is evaluated by CV and galvanostatic charge/discharge in a two-electrode configuration using the potentiost (CHI 660E).

## Additional Information

**How to cite this article**: Fan, T. *et al*. Fabricating of high-performance functional graphene fibers for micro-capacitive energy storage. *Sci. Rep.* **6**, 29534; doi: 10.1038/srep29534 (2016).

## Supplementary Material

Supplementary Information

## Figures and Tables

**Table 1:** Physical and chemical properties of functional graphene fibers.

Fibers	Mass ratio	Tensile strength (MPa)	Surface area (m^2^g^−1^)	Density of dry fiber (g/cm^3^)	Conductivity (s/cm)
GOF-a	1:16(IL:GO)	356 ± 16	238 ± 12	0.58 ± 0.03	—
GOF-b	1:8(DA:GO)	296 ± 12	166 ± 8	0.63 ± 0.03	—
GF-a	1:16(IL:GO)	476 ± 16	325 ± 14	0.56 ± 0.03	106 ± 22
GF-b	1:8(DA:GO)	365 ± 14	288 ± 12	0.57 ± 0.03	88 ± 17
GF-a annealing at 2800 °C	1:16(IL:GO)	729 ± 21	385 ± 16	0.51 ± 0.03	412 ± 32
GF-b annealing at 2800 °C	1:8(DA:GO)	632 ± 26	326 ± 18	0.54 ± 0.03	326 ± 36

(Abbrev: DA, diamine; IL, ionic liquid).
