# Supplementary material for: Fabricating of high-performance functional graphene fibers for micro-capacitive energy storage
Source: Sci Rep. 2016 Jul 8;6:29534. doi: 10.1038/srep29534 (PMC4937368; doi:10.1038/srep29534)
Supplement: Supplementary Information [file srep29534-s1.doc]

Supplementary Information for

Fabricating of high-performance functional graphene fibers for micro-capacitive energy storage

Tianju Fan^1^, Chunyan Zhao^2^, Zhuangqing Xiao^2^, Fangjun Guo^1^, Kaiyu Cai^2^, Hai Lin^1^, Yidong Liu^1^,*, Hong Meng^1^,*, Yong Min^2^,*, Arthur J. Epstein^3^

^1^ *School of Advanced Materials, Peking University Shenzhen Graduate School, Shenzhen 518055, China.*

^2^ *Institute of Advanced Materials, Nanjing University of Posts and Telecommunications, Nanjing 210046, China.*

^3^ *Department of Physics and Chemistry & Biochemistry, Ohio State University, Columbus, OH 43210, USA.*

* Corresponding authors

liuyd@pkusz.edu.cn (Y. Liu); menghong@pkusz.edu.cn (H. Meng); iamygmin@njupt.edu.cn (Y. Min)

**Contents:**

**Figure S1** AFM spectra of GO sheets;

**Figure S2** (**a**) p-phenylenediamine as coagulants in the process of spinning graphene oxide fiber; (**b**) Schematic illustrations of the formation GOF-b with diamine covalent bond with between GO layers;

**Figure S3** SEM images of twisting GOF-a to yarns;

**Figure S4** SEM images of the cross section of GF-a annealing at 2800 °C;

**Figure S5** SEM images of the cross section of GF-b annealing at 2800 °C;

**Figure S6** The FT-IR spectra of GOF-a, GOF-b and GOF-c.


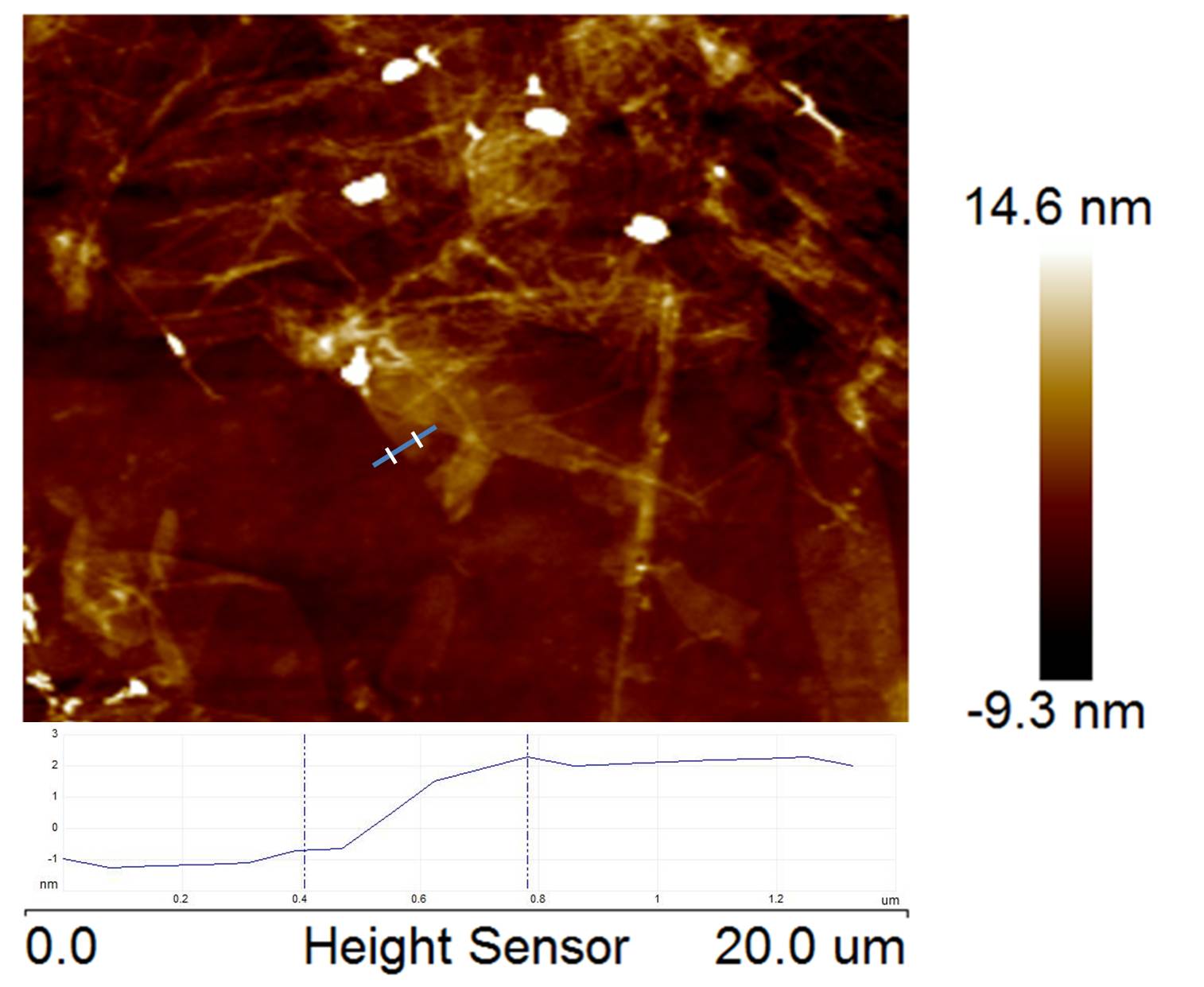


**Figure S1** AFM spectra of GO sheets.


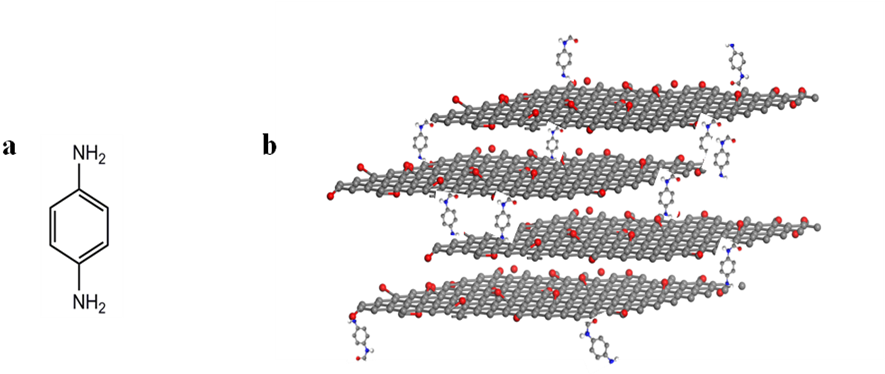


**Figure S2** (**a**) p-phenylenediamine as coagulants in the process of spinning graphene oxide fiber; (**b**) Schematic illustrations of the formation GOF-b with diamine covalent bond with between GO layers.


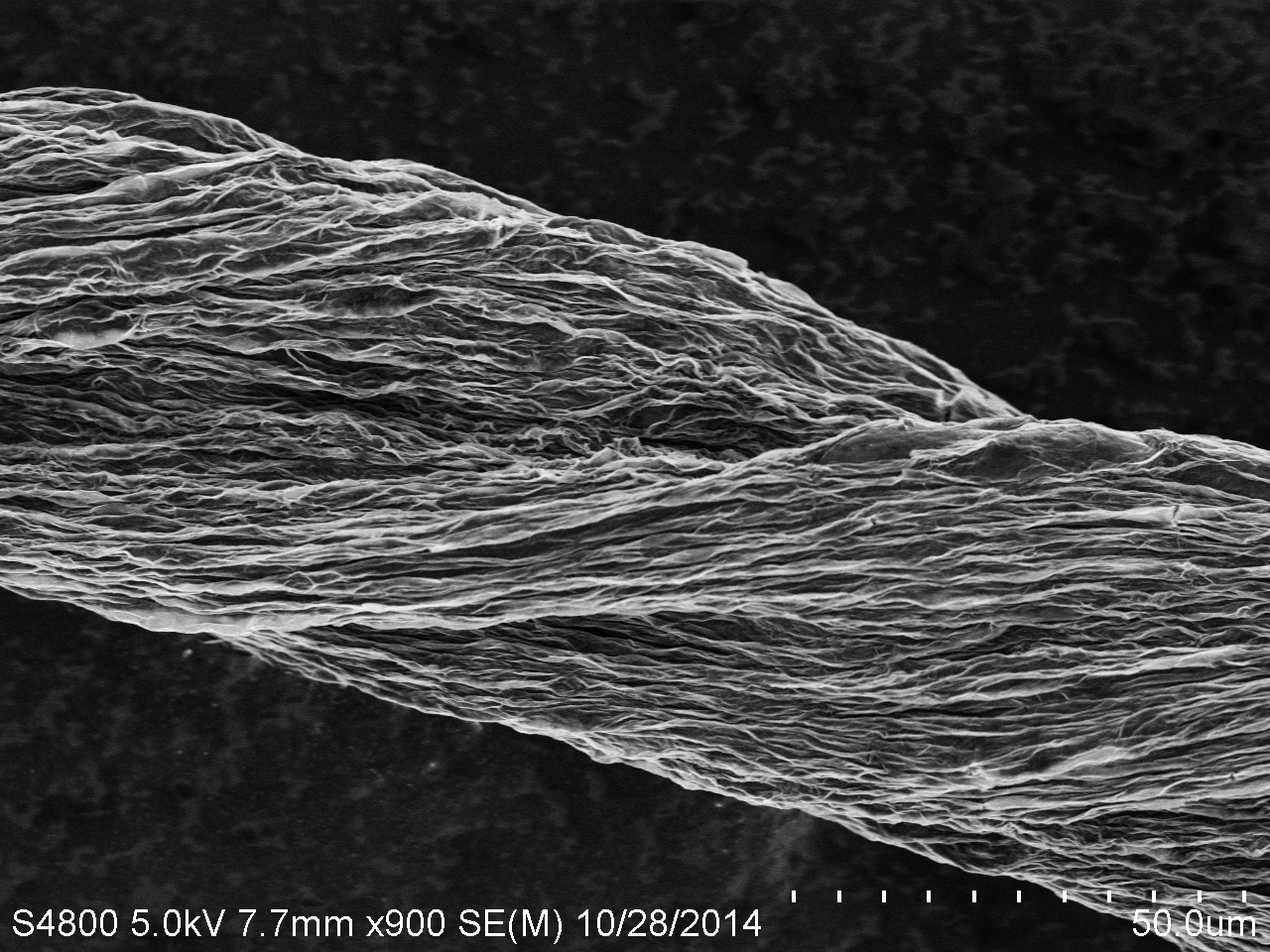


**Figure S3** SEM images of twisting GOF-a to yarns, scale bar 5 μm.


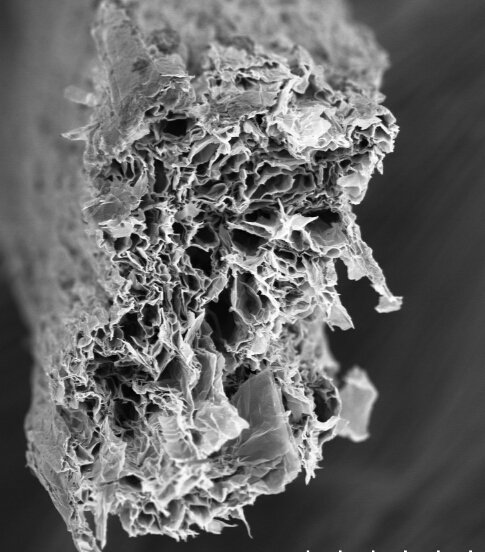


**Figure S4** SEM images of the cross section of GF-a annealing at 2800 °C, scale bar 2 μm.


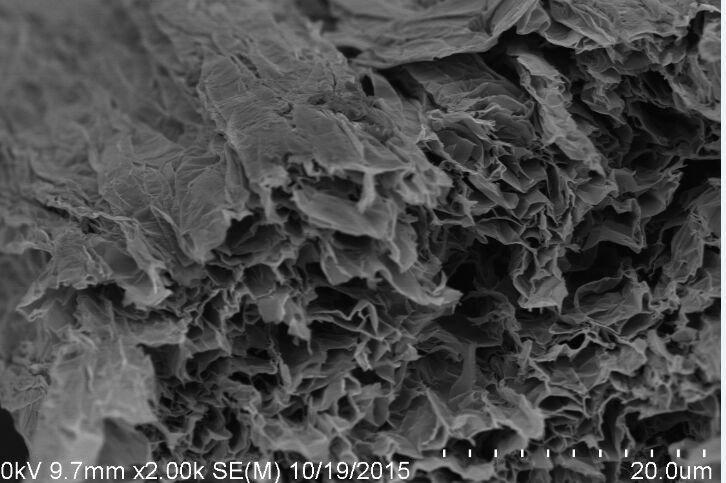


**Figure S5** SEM images of the cross section of GF-b annealing at 2800 °C, scale bar 2 μm.


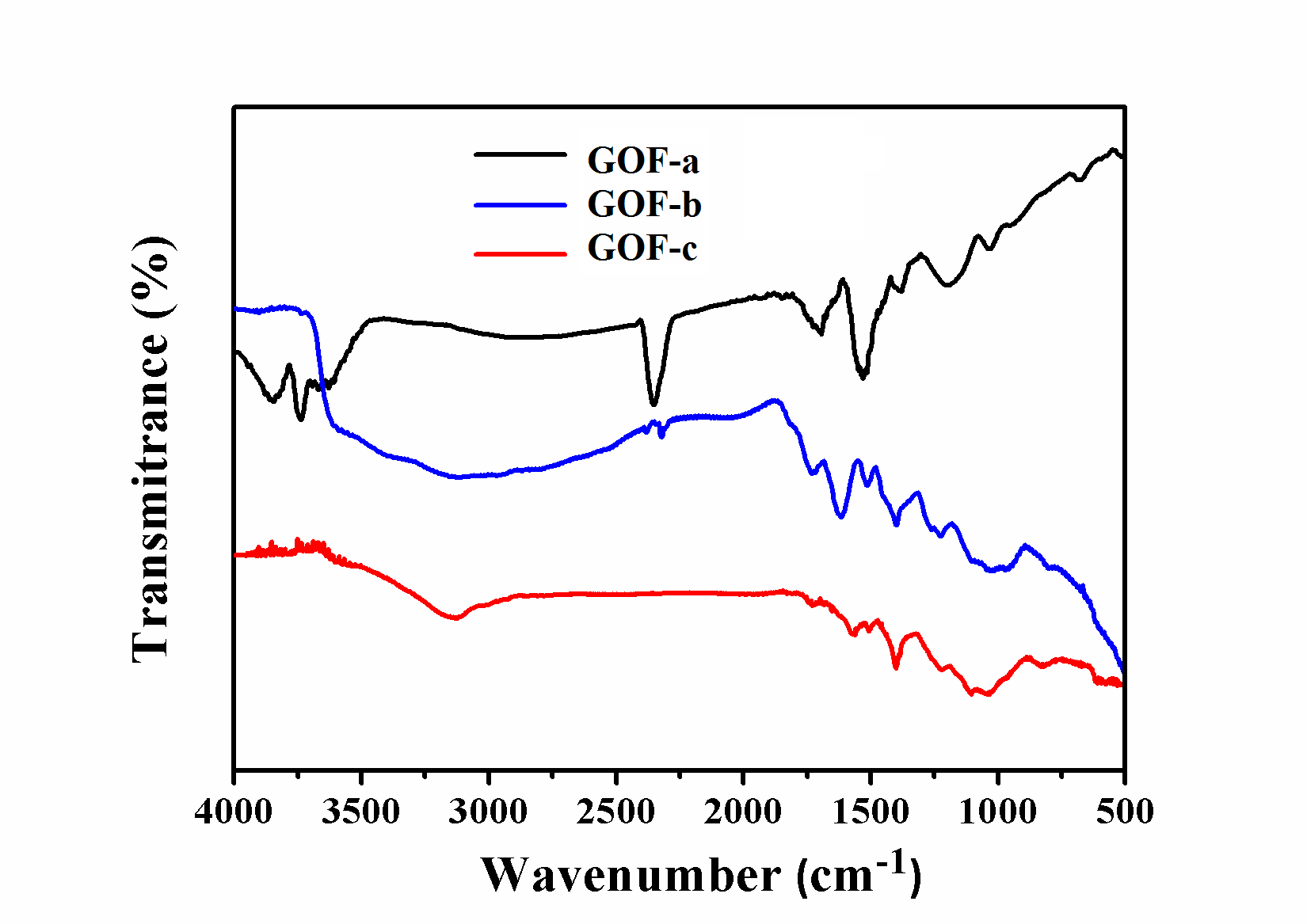


**Figure S6** The FT-IR spectra of GOF-a, GOF-b and GOF-c.
